# Septin6 and Septin7 GTP Binding Proteins Regulate AP-3- and ESCRT-Dependent Multivesicular Body Biogenesis

**DOI:** 10.1371/journal.pone.0109372

**Published:** 2014-11-07

**Authors:** Sofia Traikov, Christoph Stange, Thomas Wassmer, Perrine Paul-Gilloteaux, Jean Salamero, Graça Raposo, Bernard Hoflack

**Affiliations:** 1 Biotechnological Center, Technische Universität Dresden, Dresden, Germany; 2 School of Life and Health Sciences, Aston University, Birmingham, United Kingdom; 3 Molecular Mechanisms of Intracellular Transport Laboratory, CNRS-Institut Curie, Paris, France; 4 Cell and Tissue Imaging Facility, PICT-IBiSA & Nikon Imaging Center, CNRS-Institut Curie, Paris, France; Institut Curie, France

## Abstract

Septins (SEPTs) form a family of GTP-binding proteins implicated in cytoskeleton and membrane organization, cell division and host/pathogen interactions. The precise function of many family members remains elusive. We show that SEPT6 and SEPT7 complexes bound to F-actin regulate protein sorting during multivesicular body (MVB) biogenesis. These complexes bind AP-3, an adapter complex sorting cargos destined to remain in outer membranes of maturing endosomes, modulate AP-3 membrane interactions and the motility of AP-3-positive endosomes. These SEPT-AP interactions also influence the membrane interaction of ESCRT (endosomal-sorting complex required for transport)-I, which selects ubiquitinated cargos for degradation inside MVBs. Whereas our findings demonstrate that SEPT6 and SEPT7 function in the spatial, temporal organization of AP-3- and ESCRT-coated membrane domains, they uncover an unsuspected coordination of these sorting machineries during MVB biogenesis. This requires the E3 ubiquitin ligase LRSAM1, an AP-3 interactor regulating ESCRT-I sorting activity and whose mutations are linked with Charcot-Marie-Tooth neuropathies.

## Introduction

Septins (SEPTs) comprise a family of GTP-binding proteins that assemble into oligomers and form higher-order structures *in vitro* [1], [2]. In mammalian cells, SEPTs have been implicated in multiple cellular processes [3], [4], including cytoskeleton organization by binding to F-actin [5] or microtubules [6], [7]. In polarized cells, SEPT2 facilitates post-Golgi vesicle transport to the plasma membrane by maintaining poly-Glu microtubules [8] and forms a diffusion barrier at the base of the ciliary membrane [9], [10]. In neurons SEPT3 and SEPT5 are required for synaptic vesicle fusion and recycling [11]. During phagocytosis of pathogens, SEPT2 or SEPT9 mediates caging of bacteria to counteract pathogen dissemination [12], [13].

After endocytosis, transmembrane proteins are sorted in early endosomes to different destinations, either back to the cell surface or to the trans-Golgi network (TGN) or to late endocytic compartments for degradation. Transport from early to late endosomes is a complex membrane maturation process involving the formation of multivesicular bodies (MVBs). During this process, transmembrane cargos destined to remain in the outer membrane of late endocytic compartments such as lysosome membrane proteins (LAMPs) are segregated away from cargos destined for degradation inside maturing early endosomes. The ESCRT complex (comprising ESCRT-0, I, II and III subcomplexes) segregates ubiquinated cargos destined for degradation into vesicles budding inside maturating early endosomes [14], [15], [16], [17], [18]. The sorting of cargos destined to remain in the outer membrane is less clear. AP-3, one member of the hetero-tetrameric adaptor complexes [19], [20], localizes to peripheral early endosomes [21] and functions in the targeting of cargos destined to remain in the outer membrane of lysosomes and lysosome-related organelles [22], [23], [24], [25]. Mutations in AP-3 are linked with Hermansky-Pudlak syndrome [26]. MVB biogenesis also requires extensive membrane remodeling, in particular the exchange of the Rab5 GTPase by Rab7 controlling endosome maturation [27], [28]. It also requires membrane binding to cytoskeleton elements, in particular a switch from F-actin, which maintains early endosomes in the cell periphery [29] to microtubules needed for MVB transport to perinuclear late endocytic compartments [30].

Our previous studies identified SEPT6 and SEPT7 and their effector BORG4, a negative regulator of the Cdc42 GTPase that controls septin organization [31], among the proteins supporting AP-3 sorting function [32]. We now illustrate that SEPT6 and SEPT7 regulate MVB biogenesis by modulating the timely coordinated interaction of both AP-3 and ESCRT with maturing early endosomal membranes when bound to F-actin.

## Results

### SEPT6 and SEPT7 regulate early to late endosome transport

**Figure 1:**
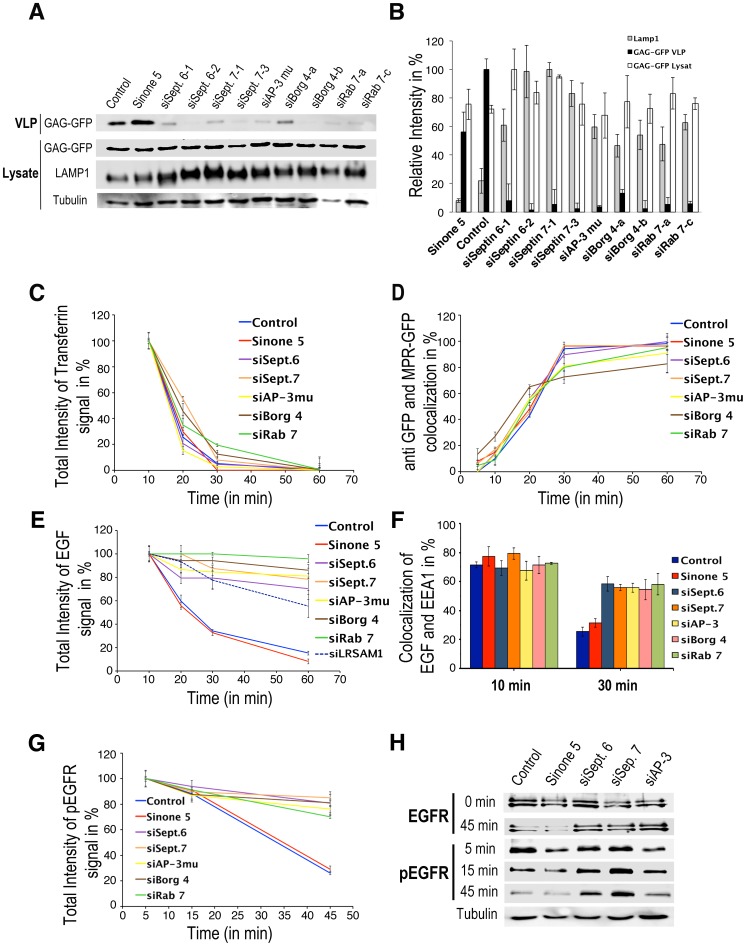
Depletion of SEPT6, SEPT7, BORG4, AP-3μ and Rab7 affect transport from early to late endosomes. (**A**) Hela expressing Gag-GFP cells were transfected with indicated siRNAs. Culture supernatants (VLP) and cell lysates prepared as described in Materials and Methods were probed by western blotting with anti GFP and anti LAMP1 antibodies and then quantified (**B**). Values were normalized to tubulin. The secreted GAP-GFP represented 59±4, 46±1, 14±7, 5.7±3, 9.8±4.5, 8.2±5, 11±5.8, 21.8±7, 10.8±7.6, 15±8.9, 14±6.5% of the total GAG-GFP. (**C**) Cell surface receptor bound Alexa 564-transferrin was endocytosed for the indicated period of time in HeLa cells treated with the indicated siRNAs. Fluorescence intensities were then quantified. (**D**) Anti GFP antibodies pre-bound to the cell surface GFP-MPR of GFP-MPR expressing cells treated with the indicated siRNAs were endocytosed for indicated periods of time as indicated in Materials and Methods. Co-localization between the endocytosed anti GFP antibody and GFP-MPR was quantified. (**E**) Cell surface receptor bound Alexa-EGF (green) was endocytosed for the indicated periods of time in HeLa cells treated with the indicated siRNAs as indicated in Materials and Methods. Fluorescence intensities were quantified. (**F**) Similarly treated cells were stained with antibodies against EEA1 and the extent of co-localization of Alexa-EGF with EEA1 was calculated. (**G**) Cell surface receptor bound EGF was endocytosed for the indicated periods of time in siRNA-treated HeLa cells. The cells were stained with antibodies against the activated EGF receptor phosphorylated on Tyr1068 (pEGFR) and EEA1. The fluorescence associated with the pEGFR was quantified. (**H**) Lysates from similarly treated cells were probed by western blotting using antibodies against the pEGFR or the total EGF receptor. The values are means ± SD of 3 independent experiments.

We previously found that SEPT6, SEPT7 or BORG4 are involved in the AP-3-dependent, lysosomal targeting of LAMP1, a protein destined to remind in the outer membrane of late endosomal compartments [32]. Besides contributing to lysosome biogenesis, AP-3 has also been implicated in HIV-1 biogenesis [33]. HIV-1 biogenesis can be examined by the release of the nucleo-capsid protein Gag into the culture medium of Gag-expressing cells. We therefore measured the release of Gag virus-like particles (VLP) from HeLa cells depleted in SEPT6, SEPT7, BORG4, AP-3 or Rab7 (≈80–95% knockdown efficiencies, see Fig. S1). Fig. 1A, B shows that the depletion of these proteins inhibited Gag VLP release into the culture medium and also resulted in a higher LAMP1 stability (i.e. a longer half-life most likely due to changes in its trafficking) in those cells. Therefore, SEPT6, SEPT7, BORG4, AP-3 and Rab7 are all required for a proper HIV-1 Gag and LAMP1 sorting.

EEs are sorting stations from where endocytosed components are sent to different destinations. Therefore, we investigated whether these pathways were affected upon SEPT6, SEPT7, BORG4, AP-3 and Rab7 depletion. We first measured the recycling of the transferrin receptor back to the cell surface after its endocytosis. Quantitative image analysis of endocytosed Alexa Fluor 564-labeled transferrin (referred to as Alexa-transferrin) (Fig. 1C, Fig. S2C) shows that recycling was mildly affected in depleted cells. To monitor the retrograde transport of endocytosed MPR back to the TGN, we took advantage of a HeLa cell line stably expressing a GFP-tagged version of MPR [34]. Quantitative image analysis of anti GFP antibodies bound to cell surface GFP-MPRs and subsequently endocytosed (Fig. S2D) shows that GFP-MPR recycling back to the TGN was also not affected in depleted cells (Fig. 1D).

We then monitored the endocytosis and the degradation of Alexa Fluor 488 EGF (Alexa-EGF) bound to its receptor (EGFR). In control cells, endocytosed EGF was detected in peripheral EEs 5–10 minutes after internalization, co-localizing with the EEA1 early endosomal marker. It was then concentrated into perinuclear, late endocytic structures (LEs) where it was degraded 30–60 min after internalization (Fig. 1E, F and Fig. S2A). Quantitative image analysis shows that this transport from EEs to LEs was remarkably delayed in depleted cells, EGF remaining in EEA1-positive EEs for longer periods of time (30–60 min) (Fig. 1F, and Fig. S2B). Similarly, the EGF receptor or its activated form detected with an antibody against phospho-tyrosine 1068 remained in EEs in depleted cells whereas it was rapidly degraded in LEs of control cells (Fig. 1G,H and Fig. S3A). Altogether, these results demonstrate that SEPT6, SEPT7, BORG4 AP-3 and Rab7 regulate transport of cargos from early to late endosomes without drastically affecting other pathways.

### SEPT6 and SEPT7 bound to F-actin regulate the motility of AP-3-positive endosomes and AP-3 dynamics

**Figure 2:**
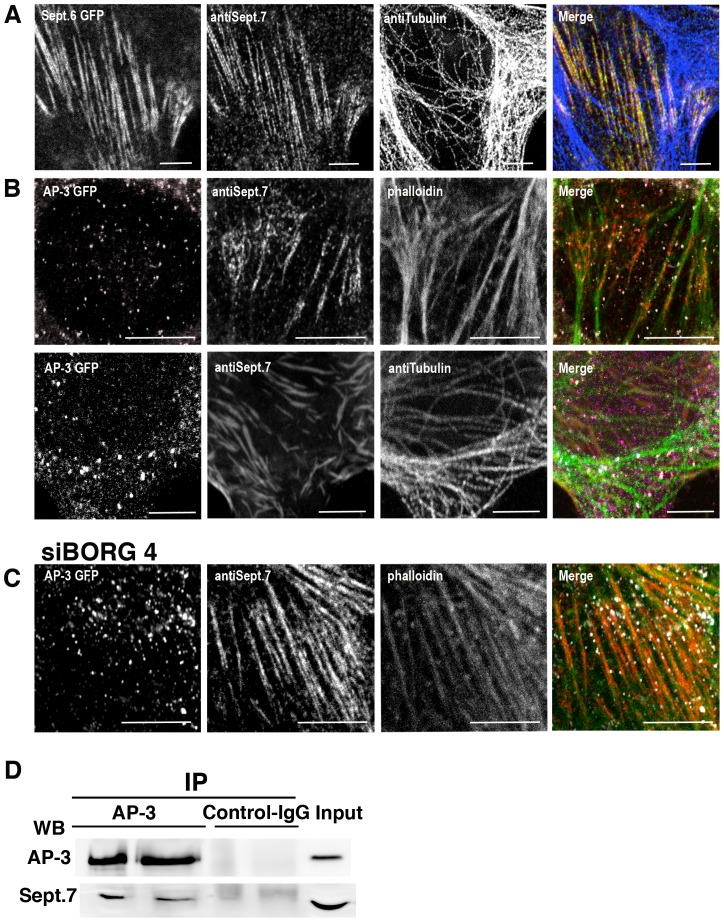
SEPT6 and SEPT7 bind F-actin and AP-3-positive structures. HeLa cells stably expressing GFP-SEPT6 (green) were labeled with anti SEPT7 (red) and anti tubulin (blue) antibodies (**A**). HeLa cells stably expressing GFP-AP3 (white) were untreated **(B)** or treated with siRNA targeting Borg4 **(C)** and then labeled with anti SEPT7 antibodies (red). F-actin and microtubules were labeled with phalloidin or anti tubulin antibodies (green) (Bar 10 µm). **(D)** AP-3 was immunoprecipitated from a HeLa cell extract with anti AP-3 antibodies and the immunoprecipitates were probed by western blotting using antibodies against AP-3 and SEPT7.

**Figure 3:**
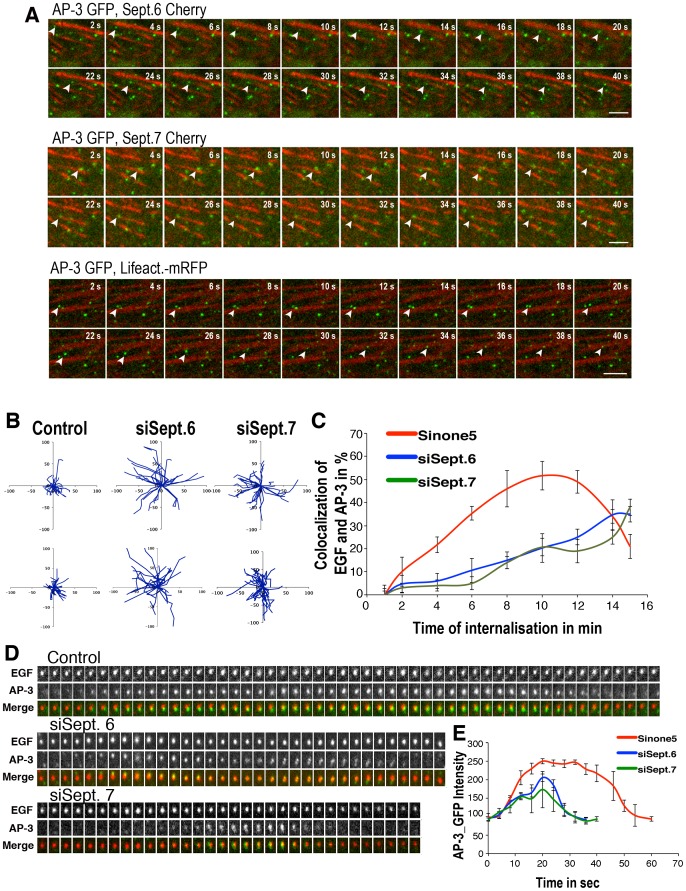
Depletion of SEPT6, SEPT7 affects the motility of endosomes and the dynamic association of AP-3. (**A**) Time-lapse video microscopy of HeLa cells stably expressing GFP-AP-3d and transiently expressing Cherry-SEPT6, Cherry-SEPT7 or mRFP-Lifeact (acquisition: 200 ms/frame, intervals between frames: 475 ms) (Bar 5 µm). (**B**) HeLa cells stably expressing GFP-AP-3δ were depleted or not from SEPT6 or SEPT7 and observed by video-microscopy. The upper panels shows examples of trajectories of GFP-AP-3δ-positive objects (300 objects per condition). Cell surface receptor-bound Alexa-EGF was endocytosed for 5 min. in control and SEPT6- or SEPT7-depleted Hela cells and Alexa-EGF-positive objects were observed by video-microscopy. The lower panels show examples of trajectories (300 objects per condition). (**C**) Cell surface bound Alexa-EGF was internalized in GFP-AP-3δ expressing HeLa cells depleted or not from SEPT6 or SEPT7 and followed by videomicrosopy. The extend of colocalization between GFP-AP-3 and Alexa-EGF was estimated. (**D**) The interaction of GFP-AP-3 with individual Alexa-EGF-positive structures was recorded and quantified (25 structures per condition) (**E**). The values are means ± SD of 3 independent experiments.

Since septin members can bind to F-actin and microtubules, we examine the precise localization of SEPT6 and SEPT7. Both were found to colocalize (Fig. 2A), as expected for complexes [35], [36] and to decorate actin filaments (85% of colocalization) (Fig. 2B). Remarkably, AP-3-positive structures were predominantly detected along F-actin decorated with SEPT7 (48.9% of AP-3-positive structures, n = 200) (Fig. 2B). Interestingly, the siRNA-mediated depletion of Borg4 enhanced SEPT7 binding onto F-actin and the association of AP-3-coated endosomes (80% of AP-3-positive structures, n = 200) with F-actin decorated with SEPT7 (Fig. 2C). These results suggested that SEPT7 mediates the interaction of AP-3-coated structures with F-actin. Accordingly, the immunoprecipitation of AP-3 from detergent-solubilized HeLa cell extracts co-immunoprecipitated significant amounts of SEPT7, thus indicating interactions between AP-3 and SEPT7 (Fig. 2E). This prompted us to follow the dynamics of AP-3-positive structures in live cells. Video microscopy performed on HeLa cells expressing GFP-tagged AP-3d and mCherry-tagged SEPT7 showed that AP-3-positive structures were immobile when bound to SEPT7 filaments, then detached moving over short distances before binding again the SETP7 filaments (Fig. 3 A, movie S1). Similar observations were made using mCherry-tagged SEPT6 or lifeact-mRFP, visualizing F-actin (Fig. 3A, movies S2, movies S3). This dynamic was drastically affected upon SEPT6 or SEPT7 depletion (movies S4, S5 and S6). Although the statistical analysis of time-lapse series indicated that the total number of motile AP-3-positive objects was not altered in control or depleted cells (not shown), it indicated that AP-3-positive objects moved along longer tracks in depleted cells when compared to control cells (Fig. 3A, upper panels, movies S4, S5, S6) with the very same speed (≈1 µm/sec) as seen in control cells. We also followed the dynamic association of GFP-AP-3 with endocytic compartments containing internalized Alexa-EGF using video microscopy. Two events could be observed: the recruitment of GFP-AP3 onto Alexa-EGF-positive EEs and the fusion of GFP-AP-3-positive structures with Alexa-EGF-positive structures. Quantitative image analysis of both events showed that a maximum co-localization (≈50%) between internalized EGF- and AP-3-postive organelles occurred 8–12 min after EGF-internalization and then decreased with time in control cells (Fig. 3C, movie S7). A more detailed analysis indicated that AP-3 remained associated to EGF-positive structures for about 1 min (Fig. 3D, E, movie S7), consistent with previous studies [37]. In SEPT6- or SEPT7-depleted cells, this dynamic was drastically affected. Image analysis showed that maximal co-localization between internalized EGF and AP-3 was delayed, occurring more than 10–15 min after EGF internalization (Fig. 3C, movies S8, S9). Further analysis showed that AP-3 remained only briefly (20–30 s) associated with EGF-positive endosomes (Fig. 3D, E, movies S8, S9). Endosomes containing EGF internalized for 5 min were also more motile upon SETP6 or SEPT7 depletion (Fig. 3B, lower panels). Thus, these experiments show that SEPT6 or SEPT7 depletion enhances the motility of AP-3-positive EEs and also reduces the dynamic interaction of AP-3 with these structures. These changes in AP-3 dynamics could explain why AP-3 cargos are missorted in SEPT6- and SEPT7-depleted cells.

### SEPT6 and SEPT7 depletion impacts the association of ESCRTI-III sub-complexes with endosomes and impairs MVB biogenesis

**Figure 4:**
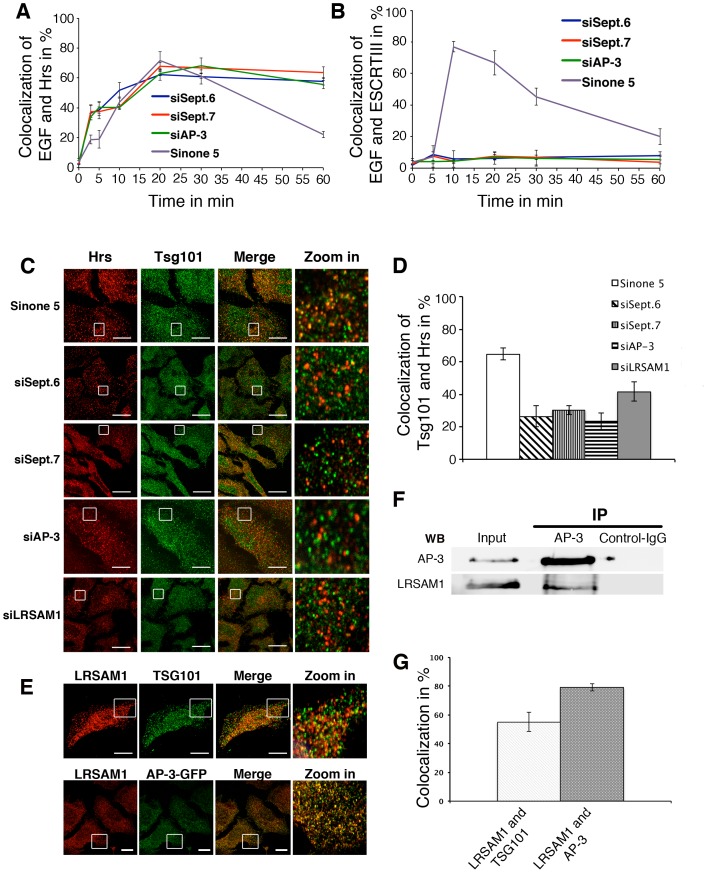
Depletion of SEPT6, SEPT7 affects the dynamic association of ESCRT sub-complexes. (**A**) Cell surface receptor-bound Alexa-EGF was endocytosed for the indicated periods of time in HeLa cells treated with the indicated siRNAs. Cells were then stained with anti Hrs antibodies. Extent of colocalization between Alexa-EGF and Hrs was quantified. (**B**) Similarly treated cells were stained with anti CHMP2B (Vps 2), an ESCRT-III subunit. Extent of colocalization between Alexa-EGF and ESCRTIII was determined. (**C**) Control and siRNA treated HeLa cells were stained with antibodies against Hrs and Tsg101. (**D**) The extent of colocalization between Hrs and Tsg101 was quantified. (**E**) Co-localization of LRSAM1 with Tsg101 and AP-3 and quantification (**G**). (**F**) AP-3 was immunuprecipitated from HeLa cell extracts with anti AP-3δ antibodies. The immunoprecipitates were probed by western blotting using antibodies against AP-3d and LRSAM1. The values are means ± SD of at least 3 independent experiments.

The ESCRT complex mediates the sorting of receptor-bound EGF into intra-luminal vesicles for degradation during MVB biogenesis. We therefore examined the association of ESCRT sub-complexes with endosomes in SEPT6, SEPT7 or AP-3 depleted cells. We first monitored the dynamic association of Hrs (Hepathocyte growth factor-Regulated tyrosine kinase Substrate) a subunit of the ESCRT-0 sub-complex [38], [39] with endosomes containing endocytosed Alexa-EGF (Fig. 4A, Fig. S3B). In control and SEPT6-, SEPT7- or AP-3-depleted cells, Hrs was detected on endosomes 5 min after EGF internalization and remained associated with these structures during the next 10–20 min (60–70% colocalization). This colocalization largely decreased in control cells after 30 min of EGF internalization, consistent with EGF degradation. In contrast, Hrs remained associated with endosomes containing endocytosed EGF in depleted cells, even 1 h after EGF endocytosis. This indicated that ESCRT-0 remains bound to EEs in the absence of SEPT6, SEPT7 or AP-3. We therefore monitored the interaction the ESCRT-III subunit CHMP2B (Vps2) with endosomes containing endocytosed EGF. In control cells, ESCRTIII was detected on EGF-rich endosomes 10–20 min after EGF internalization (up to 80% colocalization, Fig. 4B, Fig. S3C). This extent of colocalization decreased with time. In contrast, the ESCRT-III was not detected on endosomes containing endocytosed EGF in SEPT6, SEPT7 or AP-3 depleted cells. To determine which ESCRT subcomplex was affected, we examined the distribution of Tsg101, an ESCRT-I subunit. Quantitative image analysis shows that ≈60% of Hrs-positive endosomes contained Tsg101 in control cells (Fig. 4C, D). In contrast, only ≈25% of Hrs-positive endosomes contained Tsg101 in depleted cells. Altogether, these results indicate that SEPT6, SEPT7 or AP-3 depletion impacts the binding of ESCRT-I and ESCRT-III onto ESCRT-0-positive endosomes suggesting that the late stages of MVB biogenesis, i.e. formation of intra-luminal vesicles, are impaired.

**Figure 5:**
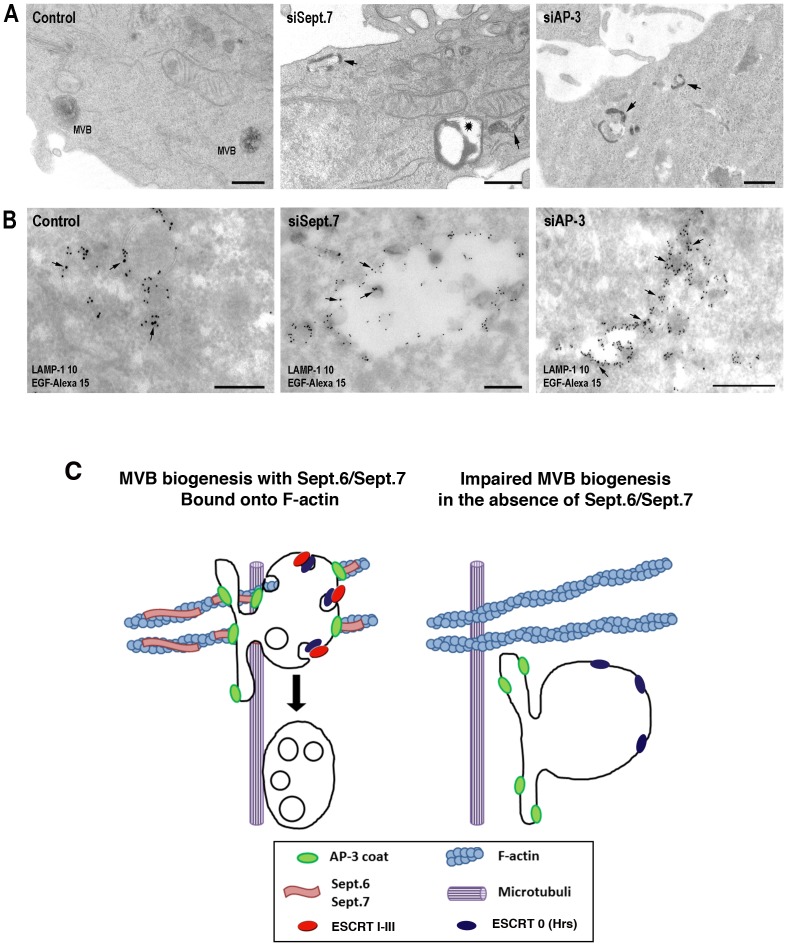
Morphology of early endosomes in SEPT7 and AP-3 depleted cells. (**A**) HeLa cells depleted or not in SEPT7 or AP-3 were allowed to fluid phase endocytose HRP for 7 min and then processed for HRP detection. (**B**). Similarly, HeLa cells were allowed to internalize for 7 min Alexa-EGF pre-bound to its cell surface receptors. Cryosections were labeled with primary antibodies against LAMP1, Alexa and secondary antibodies (LAMP-1, 10 nm gold; Alexa-EGF, 15 nm gold, arrows, Bars 200 nm). (**C**) Model of SEPT6 and SEPT7 function in MVB biogenesis.

To further examine this aspect, we used electron microscopy. HeLa cells depleted or not in SEPT7 or AP-3 were allowed to take up horseradish peroxidase (HRP) to label early endocytic structures. In control cells, HRP was detected in typical MVBs containing intraluminal vesicles (Fig. 5A). However, HRP accumulated in tubular elements and enlarged structures almost devoid of intraluminal vesicles in SEPT7-depleted cells. HRP was also detected in tubules emanating from enlarged EEs in AP-3-depleted cells. HeLa cells were also allowed to endocytosed Alexa-EGF. The examination of immuno-gold-labeled cryosections (Fig. 5B) indicated that endocytosed Alexa-EGF and low amounts of LAMP1 were detected in EEs and MVBs in control cells. However, in SEP7-depleted cells Alexa-EGF was detected in unusually enlarged structures devoid of internal vesicles and frequently found at the cell periphery, as also seen in Hrs-depleted cells [39]. The quantification of these experiments shows that SEPT7-depleted HeLa cells significantly accumulated more enlarged structures than control cells (Table 1). A 3 fold increase in LAMP1 labeling compared to controls was also detected in these structures. In AP-3 depleted cells, the morphology of EEs was also affected, exhibiting long tubular extensions rich in LAMP1, emanating from large vesicular structures almost devoid of intraluminal vesicles (Table 1), as also seen in AP-3-deficient melanocytes of patients with Hemandsky-Pudlack syndrom type II [40]. These results indicate that the late stages of MVB biogenesis are affected in SEPT7-depleted cells and more mildly in AP3-depleted cells.

**Table 1:** Quantification of MVBs and enlarged endosomes in control, SEPT7- or AP-3-depleted HeLa cells.

	MVBs (%)	Enlarged endosomes (%)
Control cells	34±8	10±7
SEPT7-depleted cells	23±6	15±5
AP3-depleted cells	24±6	22±6

HeLa cells were incubated with HRP for 7 min. and processed for electron microscopy as indicated in Materials and Methods. The labeled structures were identified as endosomal structures (vesicular and lamellar), typical MVBs and enlarged endosomes. The values ± SD represent the percentage of the indicated structures in 3 independent experiments.

### The E3 ubiquitin ligase LRSAM1 coordinates AP-3 and ESCRT sorting functions

We previously reconstituted the preferred binding of AP-3 on PI-3P-rich synthetic membranes [32] and identified by mass spectrometry LRSAM1 (leucine rich repeats and alpha sterile alpha motif containing 1) (unpublished observation), an E3 ubiquitin ligase known to ubiquitinate Tsg101, an ESCRT-I subunit [41]. LRSAM1 was found to localize better with AP-3 (80% of co-localization) than with ESCRT-I (50% of co-localization with Tsg101) (Fig. 4E,G). LRSAM1 was also found to be an AP-3 interactor as it co-immunoprecipitated with AP-3 (Fig. 4F). LRSAM1 depletion significantly delayed EGF degradation (Fig. 1E and Fig. S4), a finding, which contrasts previous findings based on over-expression [41]. Accordingly, only ≈35% of Hrs (ESCRT-0)-positive structures contained Tsg101 (ESCRT-I) in LRSAM1-depleted cells whereas ≈60% of Hrs-positive structures contained Tsg101 in control cells (Fig. 4C, D). Thus, LRSAM1 likely coordinates AP-3 and ESCRT-I sorting functions.

## Discussion

Our study demonstrates the fundamental importance of SEPT6 and SEPT7 complexes bound to F-actin during MVB biogenesis. By interacting with membrane-bound AP-3, they regulate the motility of AP-3-positive early endosomes in the cell periphery while modulating the temporal interaction of AP-3 with these structures. They also influence the membrane association of ESCRT sub-complexes during membrane maturation, a process involving the AP-3 interacting E3 ubiquitin ligase LRSAM1. Thus, our study reveals an unsuspected coordination between AP-3 sorting membrane proteins destined to remain in outer membranes of MVBs, and ESCRT sorting ubiquitinated membrane proteins into intraluminal vesicles for subsequent degradation (Fig. 5C).

Early endosomes (EEs) bind to and move along F-actin at the cell periphery [29]. The biological significance of such interactions remains unclear. Earlier studies have shown that actin polymerization inhibitors prevents the transport beyond early endosomes and leads to the formation of large vesicles, most likely unable to maturate into MVBs [42], [43]. It has been proposed that the non-processive Myosin 1B could anchor early endosomes onto F-actin [42] and that actin patches on early endosomes nucleated via Annexin2 and Spire1 control early endosome maturation [43]. We show that SEPT6 and SEPT7 bound to F-actin restrict the motility of AP-3-positive EEs at the cell periphery and influence the binding activity of AP-3 onto these structures. Remarkably,, AP-3 coats become less stable on EEs in their absence, an observation that could easily explain why Lamp1 is missorted to the cell surface in SEPT6 or SEPT7-depleted cells as previously observed in AP-3-deficient cells [23], [24], [25]. This also raises the question of how SEPT binding to F-actin could influence AP-3 binding onto membranes. Cdc42 most likely regulates SEPT6 and SETP7 binding onto F-actin as indicated by the implication of Borg4, a negative regulator of Cdc42 that control septin organization in AP-3-dependent transport. On another hand, ARF-1 regulates AP-3 binding onto membranes [44]. Therefore, one may postulate that proteins able to regulate both Cdc42 and ARF-1 activities could be involved in this particular aspect. The SEPT6/SEPT7 could exhibit addition roles in F-actin organization. It was recently reported that SEPT7 bundles F-actin filaments and induce their curvature at contractile rings during cytokenesis in drosophila [45].

Our study indicates that SEPT6 and SEPT7 also regulate the binding activity of ESCRT sub-complexes, which drive the sorting of ubiquitinated cargos into intraluminal vesicles during MVB biogenesis [15], [18]. We show that, in the absence of SEPT6 and SEPT7 ESCRT-0-positive endosomes are unable to recruit ESCRT-I and ESCRT-III to promote the formation of vesicles budding inside maturing EEs. AP-3 and ESCRT organize different membrane domains and determine their fate. However, our study indicates that AP-3 and ESCRT sorting functions are linked, a process that implicates the E3 ubiquitin ligase LRSAM1, an AP-3 interactor modulating ESCRT-I sorting function. This could provide a mechanism for a timed maturation of distinct membrane domains during this step of endocytic membrane traffic. Other E3 ubiquitin ligases have been involved in this process [18]. Therefore, the precise function of LRSAM1 remains to be fully elucidated in this specific context. It is possible that AP-3-bound LRSAM1 ubiquitinates specific cargos and/or modulates an ubiquitin-dependent ESCRT-I sorting activity. Previous studies reported that the overexpression of a LRSAM1 negative mutant LRSAM1 accelerates EGF transport and degradation [41] whereas our study shows that LRSAM1 depletion decreases EGF transport and degradation. This discrepancy can probably be explained by the methodology used (over-expression of dominant negative mutant versus gene silencing). Interestingly, mutations in LRSAM1 are linked with Charcot-Marie-Tooth neurophathies [46], so are mutations in enzymes regaling PI(3,5)P2 levels ([47], [48] and references therein) or in Rab7 {BasuRay, 2013 #104]. The Rab7-dependent maturation of EEs into MVBs also requires their interaction with microtubules [30], a process implicating the Huntington-HAP40 complex [49]. More recently, SETP6 and SETP7 have been proposed to connect F-actin and microtubule remodeling during axon branching [50]. It is therefore possible that the interaction of AP-3-coated endosomes with SEPT6 and SEPT7 bound to F-actin may also facilitate a switch from F-actin to microtubules during MVB biogenesis.

Our previous proteomic screens [32] identified a number of proteins associated with AP-3 that could coordinate both the interaction of SEPT6 or SEPT7 with F-actin and that of AP-3 with membranes or could regulate a switch from F-actin to microtubules during MVB biogenesis. Their function can now be tested in the light of our current findings.

## Materials and Methods

### Antibodies and reagents

The following antibodies were used: mouse monoclonal antibodies against human Lysosomal-associated membrane protein 1 (LAMP1) (BD Biosience), Septin6 (Abcam, UK), AP-3d (DSHB), b-Tubulin (Sigma, Germany), Early endosome antigen 1 (EEA1) (BD Biosciences), rabbit polyclonal antibodies against human Septin 7 (Sigma, Germany), BORG4 (Bethyl Laboratories, INC), GFP (Roche, Zuerich), Phosphotyrosine1068-EGF Receptor (NBG,Germany), Hrs, Tsg101 (Genetex, Germany), LRSAM1 (Atlas, Sweden). Alexa Fluor 488, 546 or 647 labeled secondary antibodies against the above-mentioned primary antibodies (Molecular Probes, Invitrogen, Germany); (HRP)-conjugated goat anti-mouse IgG and goat anti-rabbit IgG (Jackson ImmunoResearch Laboratories, UK). Phaloidin, Latrunculin B and Nocodazole were from Calbiochem (CA).

### Cell Culture, RNA interference and transfection

HeLa cells (ATCC, USA Cell Bank, UK) and HeLa cells stably expressing a GFP-tagged MPR (34) were maintained in DMEM supplemented with 10% fetal calf serum (FCS) and 1% Penicillin/Streptomycin (Invitrogen, Hamburg). HeLa cells stably expressing GFP-AP3d were generated as follows. Cells were transfected with 1 µg of GFP-AP-3d containing plasmid. Two days after transfection, cells were selected in medium supplemented with 0.8 mg/ml G418 (Gibco, Germany). After 2–3 weeks, single colonies were picked and transferred into 24-well plates. Clones were analyzed for expression by fluorescence microscopy and suitable clones were selected. For the knockdown of target genes, cells were incubated for 72 h with 20 nM siRNAs and Interferin transfection reagent (PeqLab, Erlangen) as recommended. Knockdown efficiencies were evaluated by western blot when antibodies were available or by quantitative polymerase chain reaction (QPCR) using Brilliant SYBR Green QPCR Master Mix and Mx400 QPCR System (Stratagen, Zuerich) (Figure S1). Knockdown efficiencies for SEPT6, SEPT7, AP-3, BORG4 and Rab7 were around 95%. The knockdown of one of these genes did not affect the expression of the others (Figure S1).

The siRNAs were purchased from Dharmacon Research (Chikago, IL). The following sequences (5′ -3′) were used: Septin 6 (gaagagtgctacacaccta; cgatatagctcgccaggtg), Septin 7 (ttgcagctgtgacttataa, attaagatactcttcctggac). The following siRNA were from Ambion (Foster City, CA): AP-3μ (gctcccaggtaccaaaaagTT, ggaattacagtgacagttcTT) BORG4 (cccttgattcagaccatggTT; ggaataggttttcctctgtTT) Rab7 (gcaatcagtacaaagccacTT, gctgactttctgaccaaggTT), nontargeting control (Sinone 5). LRSAM1 (ggttctagatctccacgatTT, caacggcttttgaaccagaTT).

Primer used for QPCR: BORG4-2f (gtccaggaagttccgggg), BORG4-2r (tcgaggagggggtcaggg), BORG4-3f (gtccatcatgtccttccacatc), BORG4-3r (ccggaaggcagggctgcg), Sept6-2f (ccagccacccacacacagccg), Sept6-2r (ccttactgtccagcttcttc), Sept6-3f (gactttgtgaagctgcggg), Sept6-3r (tggtgcagtttcttcagacgg), Lrsam1-f (tgtccataacccaggaggag), Lrsam1-r (atcaacggcttttgaaccag).

### Analysis of HIV-1-Gag Release

HeLa cells grown in a 6 cm plate were transfected for 42 h with different siRNA duplexes as described above. Cells were then transfected with 6 µg GAG-GFP containing vector (pEGF-n1-gag) and jetPAI reagent (PeqLab, Erlangen). The cells were incubated for 24 h and supernatant was collected, clarified by centrifugation. Membrane particles containing GAG were pelleted through ultracenrifigation in 20% sucrose layer at 60 000 rpm for 1 h and resuspended in 2× Laemli sample buffer. Cells were washed with PBS and lysed with 1% Triton X-100 containing protease inhibitor. GAG-GFP were detected by Western blot with anti GFP antibodies. For cell imaging, cells grown on coverslips were fixed with 2% PFA, permeabilized with 0.1% Triton X-100 for 5 min on ice and blocked with 3% bovine serum albumin for 30 min at RT. The cells were incubated with primary antibodies against EEA1 or LAMP1 for 1 h at RT. After washing, cells then incubated with Alexa Fluor-labeled secondary antibodies for 30 min at RT and then mounted in Mowiol.

### Assays for trafficking pathways

The HeLa cells grown on coverslips were treated with siRNAs for 72 h. They were incubated for 30 min on ice with DMEM containing 1% bovine serum albumin and 3 µg/ml EGF Alexa Fluor 488 (Molecular Probes, Germany) or 1 µg/ml Alexa Fluor 564-labeled transferrin (Molecular Probes, Germany). Cells were washed with phosphate buffered saline (PBS) and incubated at 37°C for 5, 10, 20, 30 and 60 min in DMEM containing 0.5% FCS. Cells were fixed with 3% paraformaldehyde for 15 min at RT, permeabilized with 0.1% Triton X-100 for 5 min on ice and blocked with 3% bovine serum albumin for 30 min at RT. The cells were incubated with primary antibodies against EEA1 or LAMP1 or Hrs for 1 h at RT, followed by incubation with Alexa Fluor-labeled secondary antibodies for 30 min at RT and then mounted in Mowiol. HeLa cells stably expressing MPR-GFP were treated as previously described with siRNAs and incubated with DMEM with 1% BSA and 1 µg/ml anti GFP antibodies for 30 min on ice. Cells were then washed and incubated at 37°C for 5,10,20,30 and 60 min in DMEM with 0.5% BSA. After the incubation times cells were fixed, permeabilized and blocked as previously described. The internalized anti GFP antibodies were detected with Alexa Fluor 546 secondary antibodies. Samples were analyzed by confocal fluorescence microscopy, using a confocal laser scanning microscope LSM 510 meta (Carl Zeiss Microimaging, Germany) and a 100×, 1.4 plan apochromat objective (Carl Zeiss Microimaging). Images were analyzed using Fiji software (http://rsb.info.nih.gov/ij/) or CellProfiler software. Anova Single Factor (Excel) was used for statistical image analyses.

### Live-cell imaging and quantitative analysis of vesicle dynamics

HeLa cells stably expressing GFP-AP-3d were used. Cells were treated with siRNAs as described above. They were also transfected as described above with mCherry-Septin7 or mCherry-Septin6 or pEGFP-c1-Septin6 or mRFP-Life Act plasmids. To follow the dynamic interaction of AP-3 with endocytosed Alexa-EGF, HeLa cells stably expressing GFP-AP-3 were allow to bind exogenously added Alexa-EGF on ice as described above and the cells were then washed and warmed up at 37°C and then imaged by time lapse videomicroscopy. For the live imaging, Leica AFLX6000 TIRF (with Leica HCX PL APO 100×1.40-0.70 Oil objectives) was used at a controlled temperature, CO_2_ and humidity.

GFP-AP-3-positive or Alexa-EGF-containing object trajectories were generated by wavelet segmentation and global tracking based on distance and intensity using MIA [51] was performed for all acquisition series of 600 frames. A preprocessing photobleaching correction by intensity normalization was applied to help tracking association. A coated object was considered not to move between two frames if its displacement was inferior to 3 pixels (1 pixel = 143 nm; time between two frames = 154 ms; then displacement of 1 pixel/frame = 0.93 mm/s), due to the resolution of its centroid identification. In order to exclude false tracks, only tracking trajectories of more than 5 consecutive time frames over all 600 frame series, were considered.

### Imunoprecipitation of AP-3

HeLa cells were extracted with lysis buffer (10 mM HEPES pH 7.4, 150 mM NaCl, 1 mM EGTA, 0.1 mM MgCl2, 0.5% Triton X-100) containing a complete antiprotease coktail (Roche, Penzberg). 1 mg of the cell extract proteins was incubated at 4°C for two hours with AP-3d antibodies coupled to Sepharose beads. The beads were washed 4 times with lysis Buffer+0.1% Triton-100 and incubated with Laemli sample Buffer at 90°C for 5 min. Immunoprecipitates were analyzed by Western Blot using anti AP-3d, anti SEPT7 or anti LRSAM1 antibodies.

### Electron Microscopy

For ultrathin cryosectioning and immunogold labeling, control and siRNA inactivated cells were allowed to internalize cell surface-bound EGF-Alexa (4 µm/ml) for 7 min. Cells were washed at 4°C, then fixed with a mixture of 2% PFA and 0.2% glutaraldehyde in 0.1 M phosphate buffer, pH 7.4. Cells were processed for ultracryomicrotomy and single or double immunogold labeled with the indicated antibodies detected using Protein A conjugated to gold (PAG10 or PAG15; purchased from Cell Microscopy Center, Utrecht, The Netherlands) as reported [52]. For HRP cytochemistry and conventional Electron Microscopy, control and inactivated HeLa cells grown on coverslips were grown in serum free media and allowed to internalize Horse Radish Peroxidase (HRP) (10 mg/ml, Sigma, Germany) for 7 min at 37°C. After washing at 4°C, cells were fixed with a mixture of PFA 2% and Glutaraldeyde 1.5%. Cells were washed with Cacodylate buffer 0.2M and further rinsed in Tris-HCl pH 7.6 before incubation with di-aminobenzidine and H2O2 for 20 min. Cells were further fixed with 2.5% glutaraldehyde in 0.1M cacodylate buffer for 24 h and processed as described previously [53]. All samples were analyzed using a FEI CM120 electron microscope (FEI Company) at 80 kV, and digital acquisitions were made with a numeric camera (Keen View; Soft Imaging System, SIS, Germany).

## Supporting Information

Figure S1 Efficiencies of siRNA-mediated depletions of AP-3μ, SEPT6, SEPT7, LRSAM1 and Rab7. (A) The knockdown efficiencies of the indicated **s**iRNAs were determined by qPCR as indicated in Materials and Methods. Values are means ± SD of at least 3 independent experiments. The knockdown efficiencies were confirmed by western blotting as exemplified for Borg4 (B), SEPT6 (C) and SEPT7 (D).
(TIF)

Figure S2 Endocytosis of EGF, Transferrin and GFP-MPR. In this series of experiments, only examples are provided for control, SEPT7- and AP-3-depleted cells. The quantification of these experiments is presented in Fig. 1. HeLa cells grown on cover slips were treated with siRNAs targeting SEPT6, SEPT7, BORG4, AP-3μ, Rab7 or control siRNAs. Cells were then processed as follows: (**A**) Endocytosis of EGF: HeLa cells treated with the indicated siRNAs were incubated on ice for 30 min with 5 µg/ml Alexa-EGF (green) and then incubated at 37°C for the indicated periods of time. Cells were fixed, stained with DAPI (Blue). The total fluorescence intensity of EGF-labeled objects associated per cell was then quantified. (Bars: 20 µm) (**B**) Fixed cells were also stained with antibodies against the endosomal marker EEA1 (red) and then processed for microscopy. (Bars: 10 µm). (**C**) Recycling of endocytosed transferrin: the treated HeLa cells were incubated on ice for 30 min with 1 µg/ml fluorescent transferrin and then incubated at 37°C for the indicated periods of time. Cells were fixed, stained with DAPI (Blue) and then processed for microscopy, (Bar: 20 µm). (**D**) Recycling of endocytosed GFP-MPR: Stably expressing GFP-MPR HeLa cells grown on cover slips were treated with siRNAs as above. The cells were incubated on ice for 30 min with exogenously added anti GFP antibodies and then incubated at 37°C for the indicated periods of time. Cells were fixed, stained with DAPI (Blue) and secondary antibodies against IgGs (Red), (Bar: 20 µm).
(TIF)

Figure S3 Activation of EGF receptor and interaction of ESCRT-0 and ESCRT-III with endosomes during EGF endocytosis. (**A**) Endocytosis of EGF-Receptor: HeLa cells were treated with siRNAs targeting SEPT6, SEPT7, BORG4, AP-3μ, Rab7 or control siRNAs. The cells were incubated on ice for 30 min with 5 µg/ml EGF and then incubated at 37°C for 15 min and 45 min. Cells were fixed, stained with DAPI (Blue) and antibodies against the activate form of the EGF receptor (EGFR phosphorylated on Tyr 1068, red) and the endosomal marker EEA1 (green) and then processed for microscopy (Bars 10 µm). The quantification of these experiments is presented in Fig. 1G. (**B, C**) Binding of Hrs (ESCRT-0) and CHMP2B (ESCRT-III) to endosomes containing endocytosed Alexa-EGF: HeLa cells were treated with siRNAs targeting SEPT6, SEPT7, AP-3μ or control siRNAs. (B). The cells were incubated on ice for 30 min with 5 µg/ml Alexa-EGF (Red) and then incubated at 37°C for the indicated periods of time. Cells were fixed, stained with DAPI (Blue) and antibodies against Hrs (Green) and then processed for microscopy. (C) Control and treated cells were also incubated on ice for 30 min with Alexa-EGF (Green) and then incubated at 37°C for the indicated periods of time. Cells were fixed, stained with DAPI (Blue) and antibodies against anti CHMP2B (Red). Merge images are presented (Bars 10 µm). The quantification of these experiments is presented in Fig. 4A, B.
(TIF)

Figure S4 EGF endocytosis in LRSAM1 depleted cells. HeLa cells were treated with control or siRNAs targeting LRSAM1. The cells were incubated on ice for 30 min with 5 µg/ml Alexa-EGF (Red) and then incubated at 37°C for the indicated periods of time. Cells were fixed, stained with DAPI (Blue) and then processed for microscopy. The quantification of these experiments are presented in Figure 1E.
(TIF)

Movie S1 Dynamics of GFP-AP-3-positive objects along Cherry-SEPT7 filaments (Bars 10 µm).
(AVI)

Movie S2 Dynamics of GFP-AP-3-positive objects along Cherry-SEPT6 filaments (Bars 10 µm).
(AVI)

Movie S3 Dynamics of GFP-AP-3-positive objects along mRFP-Lifeact filaments (Bars 10 µm).
(AVI)

Movie S4 Dynamics of GFP-AP-3-positive objects in control cells. The last frame of the movie represents the compilation of all frames (Bars 10 µm).
(MOV)

Movie S5 Dynamics of GFP-AP-3-positive objects in SEPT6-depleted cells. The last frame of the movie represents the compilation of all frames (Bars 10 µm).
(MOV)

Movie S6 Dynamics of GFP-AP-3-positive objects in SEPT7-depleted cells. The last frame represents a compilation of all frames (Bars 10 µm).
(MOV)

Movie S7 GFP-AP-3 dynamics during endocytosis of Alexa-EGF in control cells (Bars 10 µm).
(AVI)

Movie S8 GFP-AP-3 dynamics during endocytosis of Alexa-EGF in SEPT6-depleted cells (Bars 10 µm).
(AVI)

Movie S9 GFP-AP-3 dynamics during endocytosis of Alexa-EGF in SEPT7-depleted cells (Bars 10 µm).
(AVI)
