# Supplementary figures and images for: Septin6 and Septin7 GTP Binding Proteins Regulate AP-3- and ESCRT-Dependent Multivesicular Body Biogenesis
Source: PLoS One. 2014 Nov 7;9(11):e109372. doi: 10.1371/journal.pone.0109372 (PMC4224394; doi:10.1371/journal.pone.0109372)

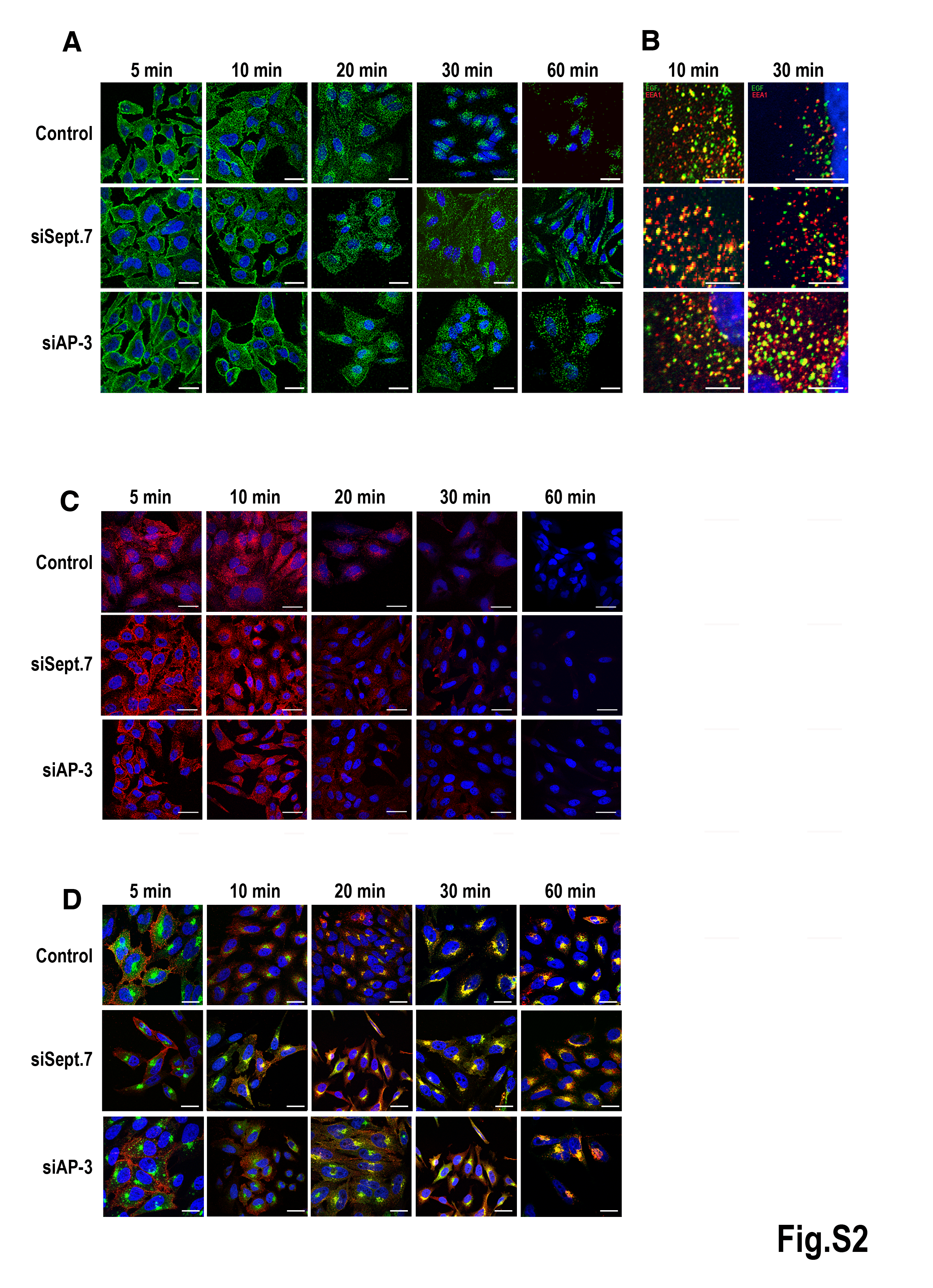

Supplement: Figure S2 — Endocytosis of EGF, Transferrin and GFP-MPR. In this series of experiments, only examples are provided for control, SEPT7- and AP-3-depleted cells. The quantification of these experiments is presented in Fig. 1. HeLa cells grown on cover slips were treated with siRNAs targeting SEPT6, SEPT7, BORG4, AP-3μ, Rab7 or control siRNAs. Cells were then processed as follows: (A) Endocytosis of EGF: HeLa cells treated with the indicated siRNAs were incubated on ice for 30 min with 5 µg/ml Alexa-EGF (green) and then incubated at 37°C for the indicated periods of time. Cells were fixed, stained with DAPI (Blue). The total fluorescence intensity of EGF-labeled objects associated per cell was then quantified. (Bars: 20 µm) (B) Fixed cells were also stained with antibodies against the endosomal marker EEA1 (red) and then processed for microscopy. (Bars: 10 µm). (C) Recycling of endocytosed transferrin: the treated HeLa cells were incubated on ice for 30 min with 1 µg/ml fluorescent transferrin and then incubated at 37°C for the indicated periods of time. Cells were fixed, stained with DAPI (Blue) and then processed for microscopy, (Bar: 20 µm). (D) Recycling of endocytosed GFP-MPR: Stably expressing GFP-MPR HeLa cells grown on cover slips were treated with siRNAs as above. The cells were incubated on ice for 30 min with exogenously added anti GFP antibodies and then incubated at 37°C for the indicated periods of time. Cells were fixed, stained with DAPI (Blue) and secondary antibodies against IgGs (Red), (Bar: 20 µm). (TIF) [file pone.0109372.s002.tif]

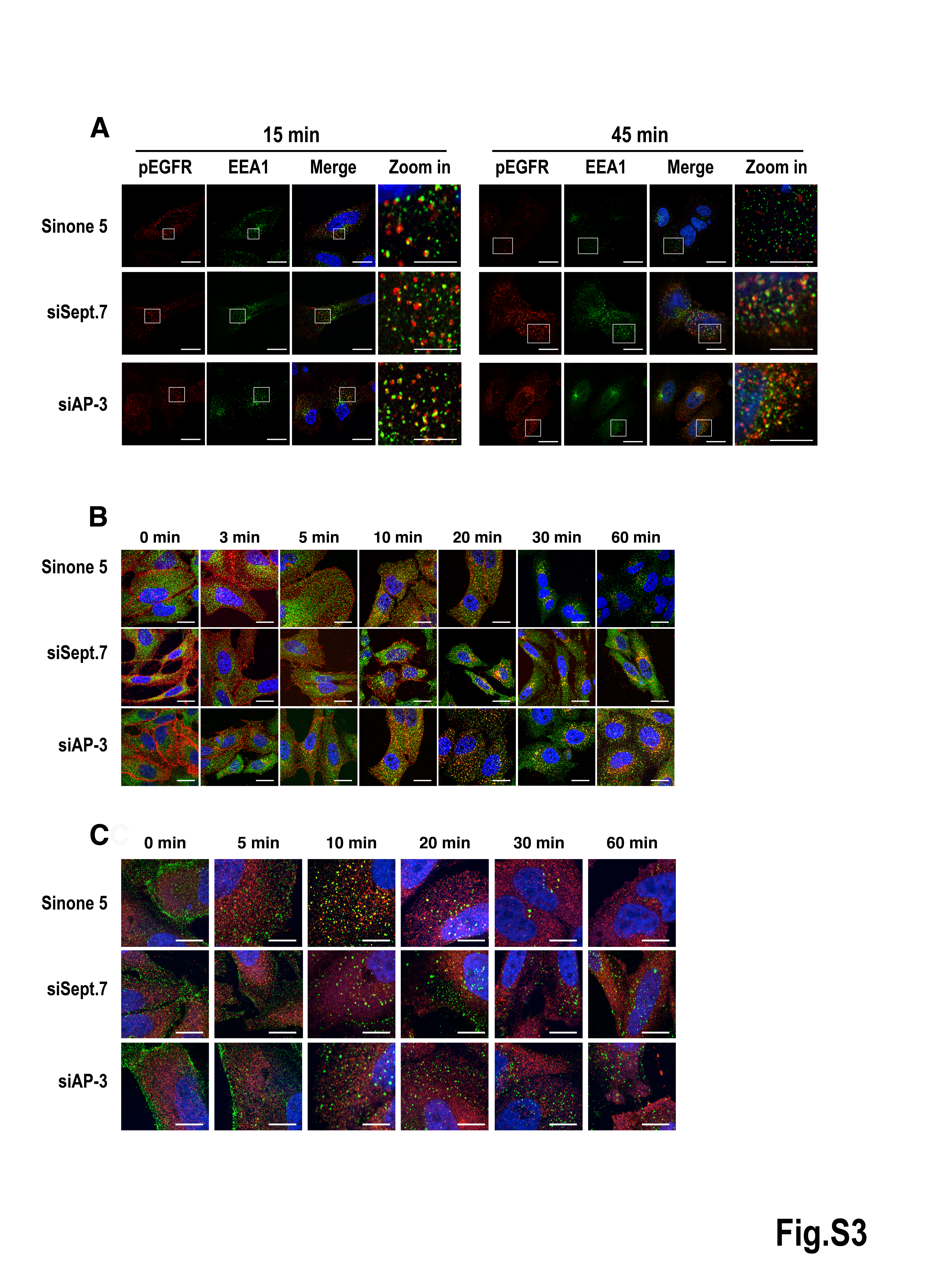

Supplement: Figure S3 — Activation of EGF receptor and interaction of ESCRT-0 and ESCRT-III with endosomes during EGF endocytosis. (A) Endocytosis of EGF-Receptor: HeLa cells were treated with siRNAs targeting SEPT6, SEPT7, BORG4, AP-3μ, Rab7 or control siRNAs. The cells were incubated on ice for 30 min with 5 µg/ml EGF and then incubated at 37°C for 15 min and 45 min. Cells were fixed, stained with DAPI (Blue) and antibodies against the activate form of the EGF receptor (EGFR phosphorylated on Tyr 1068, red) and the endosomal marker EEA1 (green) and then processed for microscopy (Bars 10 µm). The quantification of these experiments is presented in Fig. 1G. (B, C) Binding of Hrs (ESCRT-0) and CHMP2B (ESCRT-III) to endosomes containing endocytosed Alexa-EGF: HeLa cells were treated with siRNAs targeting SEPT6, SEPT7, AP-3μ or control siRNAs. (B). The cells were incubated on ice for 30 min with 5 µg/ml Alexa-EGF (Red) and then incubated at 37°C for the indicated periods of time. Cells were fixed, stained with DAPI (Blue) and antibodies against Hrs (Green) and then processed for microscopy. (C) Control and treated cells were also incubated on ice for 30 min with Alexa-EGF (Green) and then incubated at 37°C for the indicated periods of time. Cells were fixed, stained with DAPI (Blue) and antibodies against anti CHMP2B (Red). Merge images are presented (Bars 10 µm). The quantification of these experiments is presented in Fig. 4A, B. (TIF) [file pone.0109372.s003.tif]

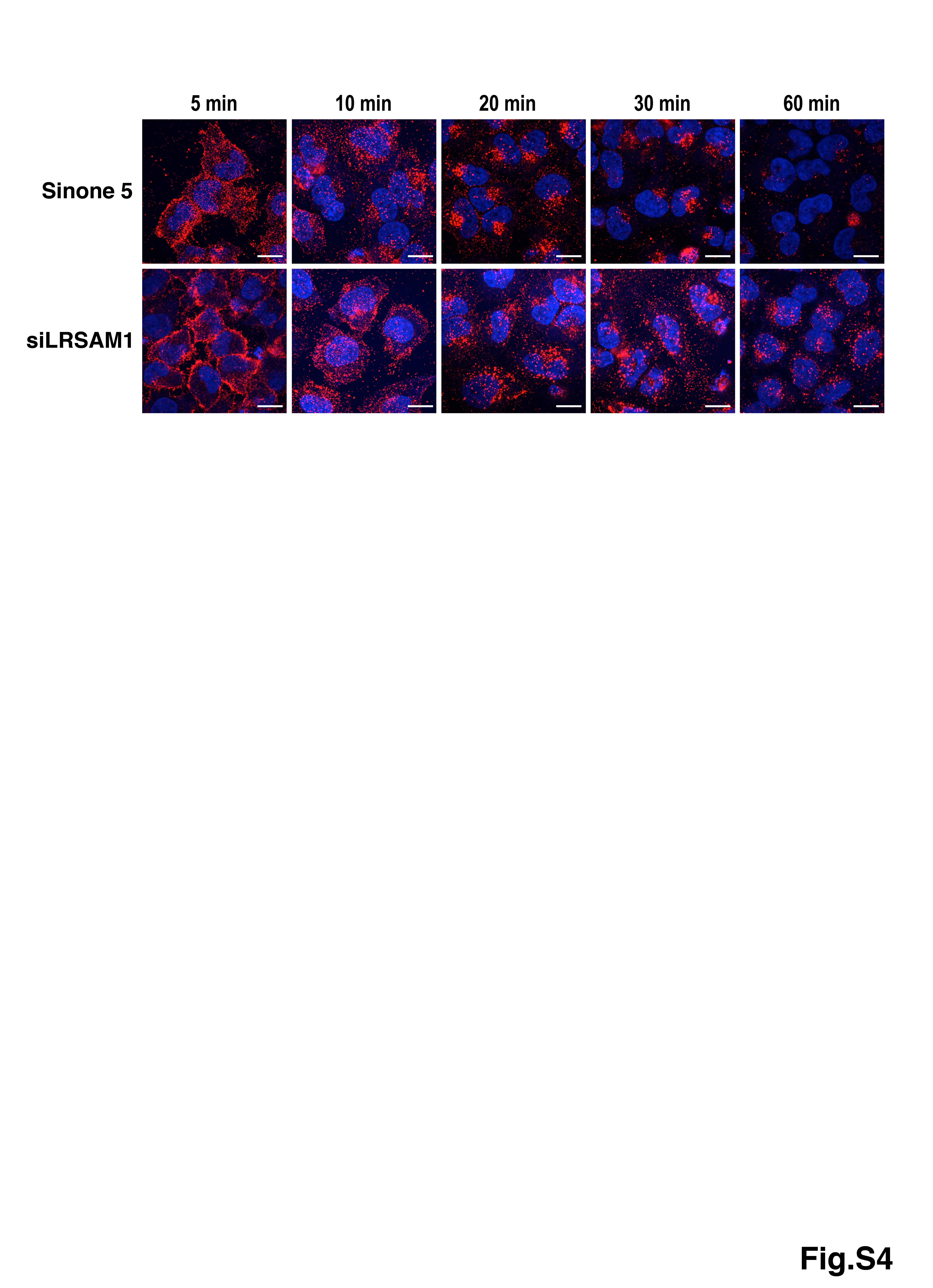

Supplement: Figure S4 — EGF endocytosis in LRSAM1 depleted cells. HeLa cells were treated with control or siRNAs targeting LRSAM1. The cells were incubated on ice for 30 min with 5 µg/ml Alexa-EGF (Red) and then incubated at 37°C for the indicated periods of time. Cells were fixed, stained with DAPI (Blue) and then processed for microscopy. The quantification of these experiments are presented in Figure 1E. (TIF) [file pone.0109372.s004.tif]
